# Reactivity of the ventromedial prefrontal cortex, but not the amygdala, to negative emotion faces predicts greed personality trait

**DOI:** 10.1186/s12993-023-00223-w

**Published:** 2023-12-01

**Authors:** Kun Deng, Weipeng Jin, Keying Jiang, Zixi Li, Hohjin Im, Shuning Chen, Hanxiao Du, Shunping Guan, Wei Ge, Chuqiao Wei, Bin Zhang, Pinchun Wang, Guang Zhao, Chunhui Chen, Liqing Liu, Qiang Wang

**Affiliations:** 1 Faculty of Psychology, Tianjin Normal University, Tianjin, 300387 China; 2 Department of Neurosurgery, Tianjin Huanhu Hospital, Tianjin, 300060 China; 3 Department of Psychological Science, University of California, Irvine, CA 92697-7085 USA; 4 Key Research Base of Humanities and Social Sciences of the Ministry of Education, Academy of Psychology and Behavior, Tianjin Normal University, Tianjin, 300387 China; 5 Tianjin Social Science Laboratory of Students’ Mental Development and Learning, Tianjin, 300387 China; 6 State Key Laboratory of Cognitive Neuroscience and Learning & IDG/McGovern Institute for Brain Research, Beijing Normal University, Beijing, 100875 China

**Keywords:** Greed, Amygdala, vmPFC, PPI, Functional connectivity

## Abstract

This study explored whether amygdala reactivity predicted the greed personality trait (GPT) using both task-based and resting-state functional connectivity analyses (n_total_ = 452). In Cohort 1 (n = 83), task-based functional magnetic resonance imaging (t-fMRI) results from a region-of-interest (ROI) analysis revealed no direct correlation between amygdala reactivity to fearful and angry faces and GPT. Instead, whole-brain analyses revealed GPT to robustly negatively vary with activations in the right ventromedial prefrontal cortex (vmPFC), supramarginal gyrus, and angular gyrus in the contrast of fearful + angry faces > shapes. Moreover, task-based psychophysiological interaction (PPI) analyses showed that the high GPT group showed weaker functional connectivity of the vmPFC seed with a top-down control network and visual pathways when processing fearful or angry faces compared to their lower GPT counterparts. In Cohort 2, resting-state functional connectivity (rs-FC) analyses indicated stronger connectivity between the vmPFC seed and the top-down control network and visual pathways in individuals with higher GPT. Comparing the two cohorts, bilateral amygdala seeds showed weaker associations with the top-down control network in the high group via PPI analyses in Cohort 1. Yet, they exhibited distinct rs-FC patterns in Cohort 2 (e.g., positive associations of GPT with the left amygdala-top-down network FC but negative associations with the right amygdala-visual pathway FC). The study underscores the role of the vmPFC and its functional connectivity in understanding GPT, rather than amygdala reactivity.

## Introduction

Fear and greed represent fundamentally divergent responses to negative stimuli. Fear, typically associated with perceived threats, often results in defensive and conservative behaviors [3, 41]. In contrast, greed, characterized by a strong desire for more and dissatisfaction with not having enough [72], can lead to aggressive [94, 106] or risky behaviors [71, 73]. This divergence is perhaps most evident within the realms of finance and economics when observing investor behaviors in response to changing market momentum [45]. For instance, fearful investors often mitigate potential losses by selling shares in struggling markets. Conversely, greedy investors often buy more shares in hopes of ‘buying low’ in anticipation of a market rebound. In other words, as greedy individuals show heightened sensitivity to negative affect [72, 106], greed may be the partial manifestation of muted fear or threat-related emotions/affect that would otherwise promote defensive and protective actions.

Beyond sharing similar behavioral roots, recent studies have suggested commonalities in the neurological bases of fear and greed, such as in the amygdala and prefrontal cortex (PFC) [3, 103, 106]. However, our basic understanding of the neurological roots of greed is less comprehensive than that of fear, especially outside of financial contexts. This paper aimed to provide an early bridge to this gap by exploring the association between brain activity in response to negative emotional stimuli and individual variability in the Greed Personality Trait (GPT). That is, we offer a simplistic, yet foundational, investigation into connecting amygdala reactivity to basic emotion regulation. Despite its limited view into the neurobiological underpinnings of emotion processing of GPT and greedy behaviors, such investigations can provide valuable insights for expanding future research [3, 41], particularly in the development of clinical interventions and treatments for relevant psychiatric disorders.

In this study, we leveraged the face-matching task, a paradigm evidenced to be highly efficient and low-cost in time for investigating basic emotional responses, such as fear and angry [50, 58, 84], without imposing potential domain-specific biases or confounds. This task allowed us to elicit brain reactivity to negative emotional faces and further explore its potential associations with greed.

### Neurological basis of greed

Converging evidence from neuroimaging and electrophysiological studies has highlighted the involvement of various brain regions, including the amygdala, medial prefrontal cortex (mPFC), lateral prefrontal cortex (lPFC), and anterior cingulate cortex (ACC), in emotion processing and regulation. The amygdala, a subcortical region, is recognized for its rapid and automatic processing of emotional stimuli, such as fearful and angry faces, as well as affective information [18, 40, 60, 61]. Aberrant activation of the amygdala has been associated with a range of psychiatric disorders. These include major depression [83], anxiety disorders [20, 38], post-traumatic stress disorder (PTSD) [64, 76], autism spectrum disorder [8, 26], borderline personality disorders [10, 53], and schizophrenia [62].

Cortical regions (e.g., mPFC, IPFC, ACC), however, exert the functions of top-down regulation to inhibit negative emotional processing in the amygdala through cognitive reappraisal and attention modulation, thereby improving individual psychological and physiological states [18, 19]. The reactivity of the amygdala to negative stimuli has been shown to predict improvements in major depression [11], depressive symptoms 2 years later [48], trait anxiety [32], cannabis use 5 years later [80], psychological vulnerability to future life stress [84], and psychological well-being [90]. Emotion-related cortical reactivity (e.g., mPFC) during tasks involving negative emotions has also been associated with anxiety and stress [105] and relapse of recurrent unipolar depression [21].

The ventral mPFC tracks the subjective value of environmental stimuli with various properties, including risk [98, 110], time [35, 101, 102], and effort [69]. This region is also thought as a core sub-region of the reward circuitry and represents primary and secondary rewards with a way of neural common currency [25, 42]. However, there is a dearth of studies investigating the potential associations between brain reactivity in the aforementioned regions involved in emotion processing/regulation and reward/valuation, and GPT. Based on consistent evidence of heightened negative emotion/affect among greedy individuals, we hypothesize similar associations. Nonetheless, the model relevant to amygdala reactivity and emotion regulation is overly simplistic and does not comprehensively capture the complexity of brain activations during emotion processing. Thus, our investigation is exploratory, aiming to uncover potential characteristics of emotional processing among greedy individuals.

Functional coupling between the amygdala and prefrontal cortex extends beyond isolated brain regions. This coupling modulates the top-down control that the prefrontal cortex exerts on the amygdala’s reactivity to negative emotional stimuli. For instance, several brain regions are commonly activated when processing fearful faces, including the bilateral amygdala, inferior parietal lobule (IPL), dorsolateral prefrontal cortex (dlPFC), and mPFC. This suggests potential functional interactions among these regions [22]. From childhood to young adulthood, the amygdala-mPFC/ACC connectivity during emotional face processing exhibits an age-related developmental pattern. This underscores the importance of amygdala-prefrontal functional coupling in the individual development of emotion processing and experiences [109].

Atypical functional interactions between the amygdala and prefrontal cortex, such as those between the amygdala and the ventromedial prefrontal cortex (vmPFC) and between the amygdala and dlPFC, have been identified as core characteristics of various emotion-related mental disorders. These include PTSD, which is associated with decreased amygdala-vmPFC functional connectivities (FC) [85, 108], autism spectrum disorder, which is also associated with decreased amygdala-vmPFC FC [85], and depression, which is associated with decreased amygdala-vmPFC/dlPFC FC [13, 96]. Furthermore, gray matter volumes (GMVs) in the vmPFC, lateral frontal pole (FPC), dlPFC, and visual pathway (i.e., lateral occipital cortex [LOC] and occipital pole [OP]) can predict individual variability in GPT [103, 106]. Based on these findings, we hypothesize that greedy individuals may also exhibit altered functional coupling between the amygdala and vmPFC/dlPFC. This could potentially explain why they manifest more negative emotions/affect.

### The present study

We employed a well-established face-matching paradigm to elicit amygdala reactivity to faces expressing negative emotions [58, 63]. We further investigated its associations with GPT in a large sample (n = 452) using both task-based and resting-state functional connectivity analyses. The face-matching task is a structured approach that has been closely associated with personality characteristics [53, 54], psychiatric disorders [51], and negative psychopathology [106]. We hypothesized that:

1. Amygdala reactivity to faces expressing negative emotions is positively correlated with individual variability in GPT. This association was tested using a region of interest (ROI) analysis on the amygdala to examine the notion of altered perception of negative emotion among greedy individuals.
2. Greedy individuals exhibit abnormal emotion regulation, subserved by the functions of the vmPFC and dlPFC, highlighting their altered emotion regulation.
3. Specific functional interactions of the amygdala- and vmPFC-based seeds with the top-down control network provide a promising understanding of the neural mechanisms underlying GPT.

## Materials and methods

### Participants

We recruited 452 college students (67.04% females, aged between 18 and 26 years old), who were separated into two Cohorts. Cohort 1 included 83 participants (78.30% females, age M ± SD = 18.76 ± 1.09) who completed a face-matching task in an MRI scanner while their functional MRI data (t-fMRI) were collected. Twenty participants in Cohort 1 were excluded due to incomplete questionnaire data (n = 8) or low-quality MRI data on head motion (framewise displacement [FD] > 0.5 mm, n = 12). Cohort 2 included 369 participants (64.50% females, age M ± SD = 19.99 ± 1.48) who only completed the resting-state fMRI scanning (rs-fMRI). Forty-three participants in Cohort 2 were excluded due to incomplete questionnaire data (n = 1) or low-quality MRI data on head motion (n = 42). No participant self-reported any history of neurological or psychiatric issues. Written informed consent was obtained from all participants before starting formal research procedures. This study was approved by the Institutional Review Board of Tianjin Normal University.

### Assessment of greedy personality trait

GPT was measured with the 7-item Dispositional Greed Scale (DGS) which has been demonstrated to yield high reliability and validity [56, 72, 99]. Participants indicated the extent to which they agreed with each item presented in the scale (e.g., “One can never have too much money”). All items were rated on a 5-point Likert scale (1 = *Strongly Disagree*, 5 = *Strongly Agree)* and total scores ranged from 7 to 25, ω_cohort1_ = 0.71, ω_cohort2_ = 0.74. Higher scores represented a higher level of greedy personality.

### Face-matching task

Participants completed a classical face-matching task to elicit amygdala reactivity [58, 84]. The task included three blocks of face matching interleaved with two blocks of a shape-matching sensorimotor control task. During the face-matching blocks, participants viewed a trio of faces and selected one of two faces (on the bottom) matching the target face (on top). Each face block consisted of one of the following expressions: fearful, angry, or neutral. Each trial in the face-matching blocks lasted 4 s with a variable interstimulus interval (ISI) of 2-6 s (Mean = 4 s), for a total block length of 82 s. In the control blocks, each of the six shape trios was presented for 4 s with a fixed ISI of 2 s, for a total block length of 42 s. Total task time was 414 s.

### Brain imaging and data acquisition

Whole-brain image data were collected using a Siemens 3 T Prisma scanner with a 64-channel head coil at the Center for MRI Research of Tianjin Normal University for both cohorts. Participants laid supine on the scanner bed and viewed visual stimuli back-projected onto a screen through a mirror attached to the head coil in a face-matching task. Foam pads were used to assist in minimizing head motion. For Cohort 2, high-resolution T1-weighted structural images were acquired using MP-RAGE sequence with the following parameters: repetition time (TR) = 2530 ms; echo time (TE) = 2.98 ms; multi-band factor = 2; flip angle = 7 degrees; field-of-view (FOV) = 224 × 256 mm^2^; slices = 192; voxel size = 0.5 × 0.5 × 1 mm^3^; The T2*-weighted resting-state functional images used the following parameters: TR = 2000 ms; TE = 30 ms; multi-band factor = 2; flip angle = 90 degrees; FOV = 224 × 224 mm^2^; slice thickness = 2 mm; slice gap = 0.3 mm; voxel size = 2 × 2 × 2mm^3^. For Cohort 1, the task-based functional images used the same parameters as Cohort 2 with the exception that the slices were tilted ~ 30 degrees clockwise from the AC-PC plane to obtain better signals in the orbitofrontal cortex.

### Task-based functional MRI preprocessing and statistical analysis in Cohort 1

The FMRI Expert Analysis Tool was used to process functional imaging data and corresponding statistical analyses in Cohort 1 [33]. The scanner automatically discarded the first four volumes for T1 equilibrium before the task. The remaining images were then realigned to correct for head movements. Data were spatially smoothed using a 5 mm full width at half maximum Gaussian kernel to balance signal-to-noise and spatial specificity while ensuring comparability and consistency of our findings with existing research [58, 84]. Data were also filtered in the temporal domain using a nonlinear high-pass filter. EPI images were first registered to the MPRAGE structural images and then into MNI standard space, using affine transformations. Registration from MPRAGE structural images to standard space was further refined using FNIRT nonlinear registration [4]. Statistical analyses were first performed in the native image space, with the statistical maps normalized to the standard space before higher-level analysis.

The data were modeled at the first level using a general linear model within FSL’s FILM module. Four category regressors were included during the face-picture period: (1) the fearful regressor; (2) the angry regressor; (3) the neutral regressor; and (4) the shape regressor. All regressors then convolved with the double-gamma canonical hemodynamic response function. Finally, the contrast analyses (i.e., Fearful + angry faces > shapes) utilized a random-effect model for group analysis and regression analysis for each individual’s GPT scores using a FLAME1 model. Group images were thresholded using cluster detection statistics, with a height threshold of z > 3.1 and a cluster probability of p < 0.05, corrected for whole-brain multiple comparisons using Gaussian Random Field Theory.

### Task-based psychophysiological interaction (PPI) analysis in Cohort 1

We utilized a generalized form of context-dependent psychophysiological interaction analysis (PPI) to systemically investigate the functional coordination between various brain regions and the vmPFC/amygdala seeds. For the vmPFC seed, we selected only the region that showed a significant correlation with GPT scores in the face-matching task as a mask. For the bilateral amygdala seed, the conjunction region between the contrast of fear + angry faces > shape in the task and the structural atlas (i.e., Harvard–Oxford atlas with a resolution of 2 mm) was defined as the mask. Participants were divided into two groups based on the median GPT score. Fifteen participants with the exact median GPT score were excluded, resulting in a final sample of 71 (n_high_ = 33, n_low_ = 38).

The time course of each seed region in fearful and angry face conditions was defined as the physiological variable. Its interactions with group labels (e.g., high vs. low group) were defined as the psychophysiological interaction variable. Regressors with no response trials and six motion parameters for head movement were also included as regressors of no interest. These analyses were performed under different conditions (Fearful or Angry faces vs. Neutral faces) and conducted with a group difference comparison between high and low groups on distinct conditions. Group images were thresholded with a height threshold of z > 3.1, and a cluster probability of p < 0.05, corrected for whole-brain multiple comparison (FWE) using Gaussian Random Field Theory.

### Resting-state functional MRI preprocessing and statistical analysis in Cohort 2

The resting-state fMRI data in Cohort 2 were first preprocessed using the GRETNA software and the AFNI toolbox [14]. The scanner automatically discarded the first eight volumes to ensure signal equilibrium. The first ten EPI volumes were also excluded for each subject after considering the scanner’s environment adaptation and issues of the magnetization disequilibrium. The remaining 290 volumes were further slice-timing corrected, realigned, and registered to the standardized MNI space. The temporal detrending, nuisance regression, and bandpass (0.01 ~ 0.1 Hz) filtering were conducted with AFNI tools. Specific details of the methodology can be found in recent previous studies [34, 99]. Participants with excess head motion (FD > 0.5 mm) were excluded. To control for the influences of head motion, we also included FD as a confounding variable in the following analyses.

First, the seed-based (e.g., vmPFC and bilateral amygdala) resting-state functional connectivity (rs-FC) maps preprocessing was conducted. We extracted the BOLD time course from each above-mentioned seed region and then calculated the correlation coefficients between that time course and the time courses from whole-brain voxels. The correlation coefficients were further converted to a normal distribution through Fisher’s Z transform and then performed the following analyses.

Second, a mixed-effect FLAME 1 model implemented in FSL was used to examine the associations between vmPFC/amygdala-seed-based rs-FC and GPT. Specifically, the model incorporates both fixed effects and random effects. The benefit of this approach stems from its ability to model and estimate variances for different groups in the model, capturing potential associations between different variables. Maternal education, paternal education, age at MRI scan, gender, and FD were included as covariates. In the regression analyses, covariates were entered into the first block of equations. In the second block, mean-centered GPT was entered. Corrections for multiple comparisons were conducted at the cluster level using Gaussian Random Field Theory (voxel-wise threshold z > 3.1 and cluster-wise FEW corrected p < 0.05) [17, 34].

## Results

### Demographics

Table 1 provides demographic information in both Cohorts and their group comparisons. In Cohort 1 (n = 83), the GPT scores ranged from 13 to 33 (*M* ± *SD* = 21.69 ± 3.96) with no significant difference between genders (*t*_*(81)*_ = 1.19, *p* = 0.238). GPT did not correlate with age (*r* = 0.076, *p* = 0.497), maternal education level (*r* = − 0.072,* p* = 0.518), or paternal education level (*r* = 0.075, *p* = 0.499). In Cohort 2 (n = 369), the M ± SD of GPT was 22.70 ± 4.59. A significant gender difference was found in GPT (*t*_*(367)*_ = 2.80, *p* = 0.005) and GPT was not correlated with age (*r* = 0.011, *p* = 0.834), maternal education level (*r* = 0.027, *p* = 0.609) or paternal education level (*r* = 0.102, *p* = 0.050). In comparing Cohorts 1 and 2, significant group differences were observed in age (*t*_*(450)*_ = − 7.136, *p* = 3.873e-12), gender ratio (χ^2^ = 5.852, *p* = 0.016), and GPT scores (*t*_*(450)*_ = − 2.604, *p* = 0.010).

**Table 1:** Demographics and GPT scores

Measures	Cohort 1 (n = 83)	Cohort 2 (n = 369)	t/χ^2^	*p*
Gender (Male/Female)	18/65	131/238	5.852	0.016
Age (M ± SD)	18.76 ± 1.09	19.99 ± 1.48	− 7.136	3.87e-12
Paternal education (%)			7.839	0.250
Less than primary school	16.9	13		
Junior high school	34.9	38.8		
Vocational high School	12	15.7		
Senior high school	8.4	12.5		
Junior college education	13.3	8.9		
Undergraduate level	8.4	10.8		
More than graduate level	6	0.5		
Maternal education (%)			7.916	0.244
Less than primary school	24.1	19.8		
Junior high school	28.9	33.9		
Vocational high School	14.5	12.2		
Senior high school	9.6	14.6		
Junior college education	4.8	10.6		
Undergraduate level	16.9	8.9		
More than graduate level	1.2	0		
GPT (M ± SD)	21.69 ± 3.96	22.7 ± 4.59	− 2.604	0.010

*M* mean score, *SD* standard deviation

### vmPFC but not amygdala reactivity to negative emotion faces links to GPT in Cohort 1

**Fig. 1:**
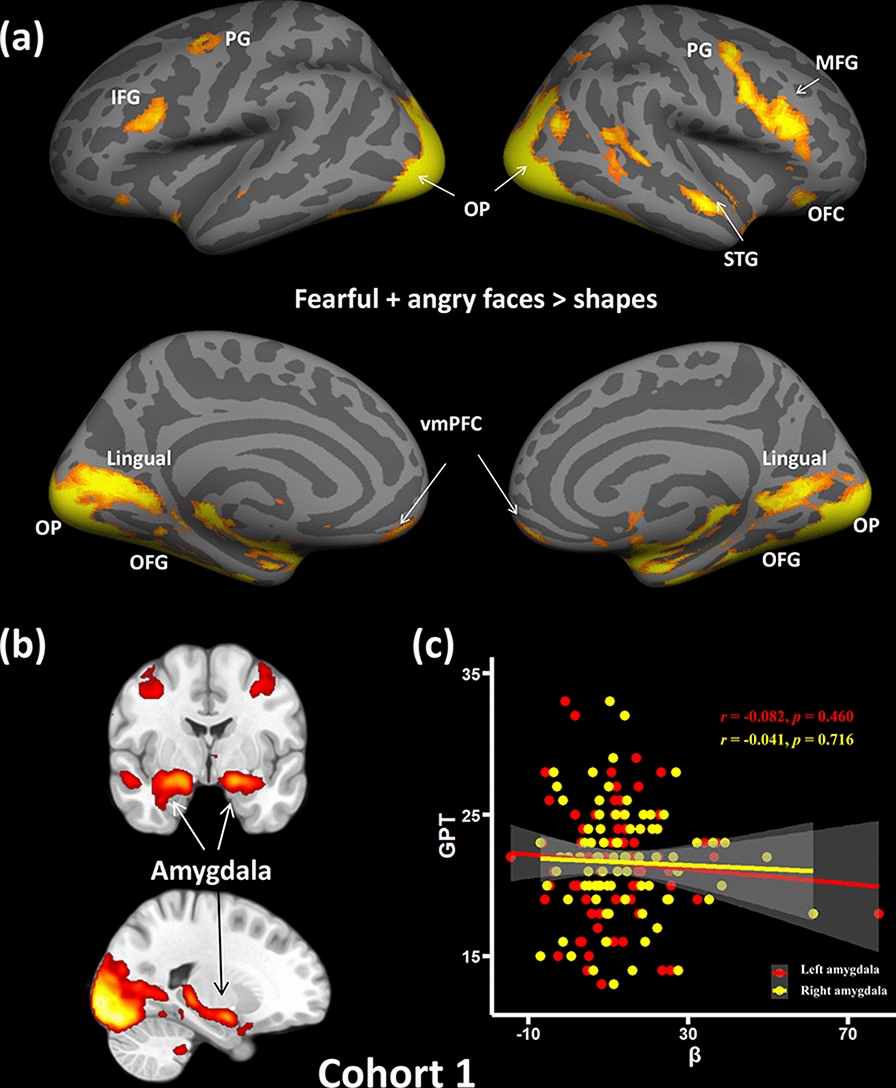
Brain regions responding to emotional faces and their associations with GPT in Cohort 1. **a** Whole-brain analysis revealed significant brain activations to negative emotion faces (Fearful + angry faces > shapes), especially including bilateral amygdala. **b** The bilateral amygdala displayed via fslview software showed strong activations when processing the negative emotion faces (Fearful + angry faces > shapes). **c** ROI analysis indicated no significant correlation between brain reactivity to negative emotion faces in the bilateral amygdala and the GPT. *vmPFC* ventromedial prefrontal cortex, *OFG* occipital fusiform gyrus, *IFG* inferior frontal gyrus, *MFG* middle frontal gyrus, *OP* occipital pole, *STG* superior temporal gyrus, *OFC* orbitofrontal cortex

The present study investigated the associations between amygdala reactivity and GPT in Cohort 1. Firstly, t-fMRI analyses were used to ensure that the face-matching task could successfully elicit predicted activation in the amygdala in Cohort 1. The contrast of fearful and angry faces > shapes was significantly associated with bilateral amygdala reactivity (left amygdala: peak MNI coordinate: *x* = − 18, *y* = − 6, *z* = − 14, *Z* = 8.80; right amygdala: MNI: *x* = 26, *y* = − 2, *z* = − 16; *Z* = 8.19) (Fig. 1a, b). Other brain regions showing similar patterns included the vmPFC, middle frontal gyrus [MFG], frontal pole [FP], inferior frontal gyrus [IFG], lateral occipital cortex [LOC], occipital fusiform gyrus [OFG], superior temporal gyrus [STG], orbitofrontal cortex [OFC], bilateral hippocampus, bilateral lingual, bilateral precentral gyrus, and cerebellum (Fig. 1a). Additional details are given in Table 2. A mean parameter estimate reflecting amygdala reactivity as a function of our task (i.e., fearful and angry facial expressions versus shapes) was extracted for each subject and used in additional correlational analyses. However, we did not observe any associations between amygdala reactivity to threat and GPT (left amygdala: *r* = − 0.082; *p* = 0.460; right amygdala: *r* = − 0.041 *p* = 0.716; Fig. 1c) and even after controlling for several covariates such as parental education, age, gender, and FD (both *p* values > 0.573).

**Table 2:** Brain region activations responding to negative emotion faces and their links with GPT in Cohort 1

Measure	Brain region	Cluster size (voxels)	MNI coordinates			Z
			*x*	*y*	*z*	
F + A > S	L Amygdala	216	− 18	− 6	− 14	8.80
	R Amygdala	269	26	− 2	− 16	8.19
	R Frontal Pole/vmPFC	392	4	54	− 14	5.09
	R Middle Frontal Gyrus	1711	52	20	30	5.71
	L Inferior Frontal Gyrus	295	− 40	14	26	4.69
	R Lateral Occipital Cortex	2046	22	− 94	− 10	12.50
	L Occipital Fusiform Gyrus	254	− 28	− 84	− 16	12.01
	R Occipital Fusiform Gyrus	268	24	− 86	− 16	11.88
	R Superior Temporal Gyrus	278	50	− 8	− 16	5.51
	L Middle Frontal Gyrus	252	− 34	− 2	48	4.84
	R Orbitofrontal Cortex	162	34	32	− 14	5.59
	L Orbitofrontal Cortex	34	− 40	26	− 14	4.06
	L Hippocampus	433	54	60	29	8.8
	R Hippocampus	550	36	62	28	8.19
	L Lingual	1177	59	19	28	12
	R Lingual	1147	34	16	31	12.5
	L Precentral gyrus	241	62	62	60	4.84
	R Precentral gyrus	528	24	67	51	5.71
	R cerebellum	154	2	− 54	-34	5.19
F + A > S & GPT )(-)	R vmPFC	104	2	50	0	4.26
	R Supramarginal Gyrus	64	68	− 38	20	3.81
	R Angular	32	44	− 46	20	4.02
	L Cerebellum	32	− 38	− 56	− 42	3.64
	R Superior Temporal Gyrus	32	62	2	− 4	3.98
A > N & GPT(-)	L vmPFC	49	− 8	34	− 10	3.72
	L Medial Frontal Cortex	33	− 2	44	− 20	3.55
F > N & GPT (-)	L vmPFC	104	− 4	50	− 12	3.68
	R Cingulate Cortex	38	12	− 46	30	3.51
	R Cerebellum	31	28	− 74	− 32	3.60

Significant brain regions were presented on the contrast of fearful + angry faces > shapes in Cohort 1. Brain reactivity on the contrasts of fearful + angry faces > shapes, angry > neutral faces, and fearful > neutral faces, were positively correlated with GPT in Cohort 1.

*R* right, *L* Left, *vmPFC* ventromedial prefrontal cortex, *F* Fearful face, *A* Angry face, *N* Neutral face, *GPT* greed personality trait,   - negative correlation between GPT and brain activation

**Fig. 2:**
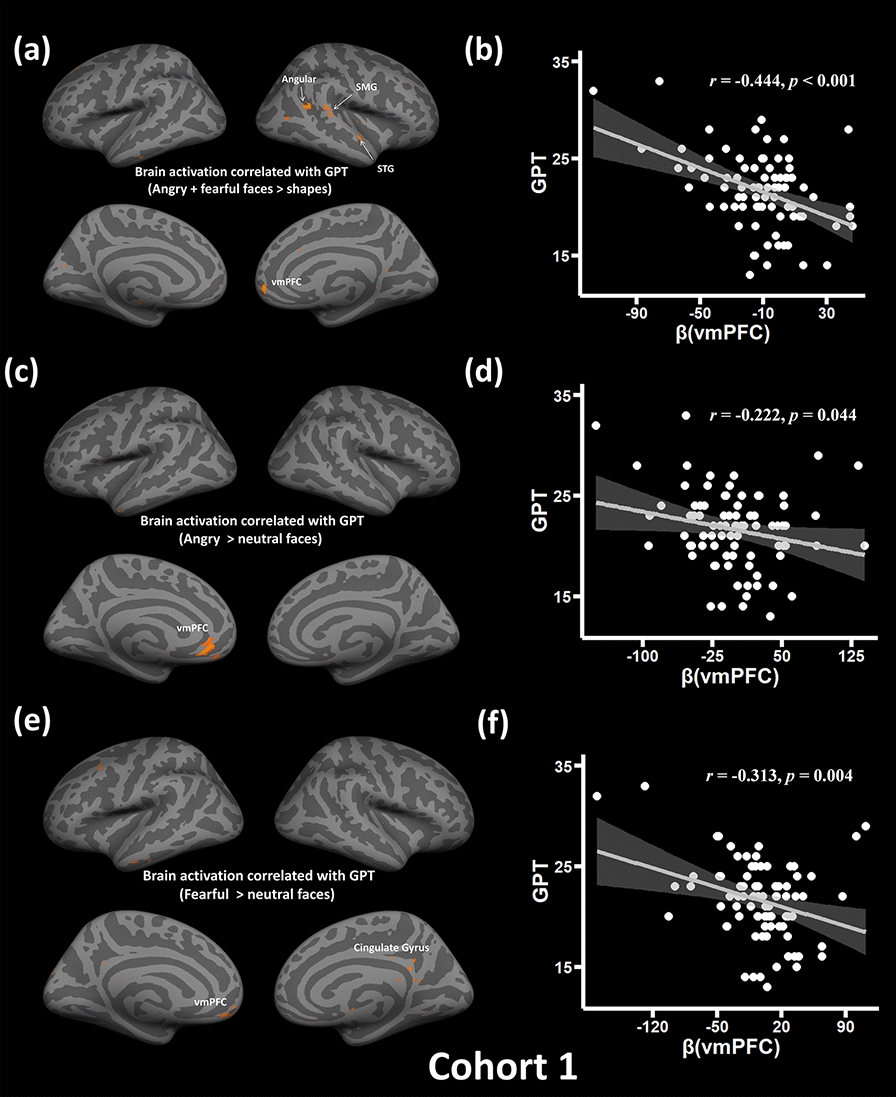
Brain region activations responding to negative emotion faces associated with GPT in Cohort 1. Brain reactivity to negative emotion faces in the vmPFC was negatively correlated with GPT in the contrast of fearful + angry faces > shapes (**a**), angry > neutral faces (**c**), and fearful > neutral faces (**e**), and corresponding scatter plots between GPT scores and activation coefficient in vmPFC (β) (**b**, **d**, **f**). All conditions revealed negative correlations between the vmPFC reactivity to negative emotion faces and GPT scores. *vmPFC* ventromedial prefrontal cortex, *GPT* greed personality trait

Secondly, we performed exploratory analysis on the associations between brain activations (i.e., Fearful + angry faces > shapes) and GPT at the whole-brain level in Cohort 1. GPT negatively varied with several brain activations, including the right vmPFC (*xyz* = 2, 50, 0; Cluster size = 104; *Z* = 4.26), right supramarginal gyrus (SMG; *xyz* = 68, − 38, 20; Cluster size = 64; *Z* = 3.81), and right angular (*xyz* = 44, − 46, 20; Cluster size = 32; *Z* = 4.02) (Fig. 2a, b). Similar patterns were found in the remaining contrasts, including the fearful > neutral faces, and angry > neutral faces. In particular, vmPFC brain activations in the contrast of angry > neutral faces (*xyz* = -8, 34, -10; Cluster size = 49; *Z* = 3.72; Fig. 2c, d) and fearful > neutral faces (*xyz* = − 4, 50, − 12; Cluster size = 104; *Z* = 3.68; Fig. 2e, f) was negatively correlated with the GPT. Other regions showing similar trends are given in Table 2.

### vmPFC-related task-based and resting-state functional connectivity and GPT

**Fig. 3:**
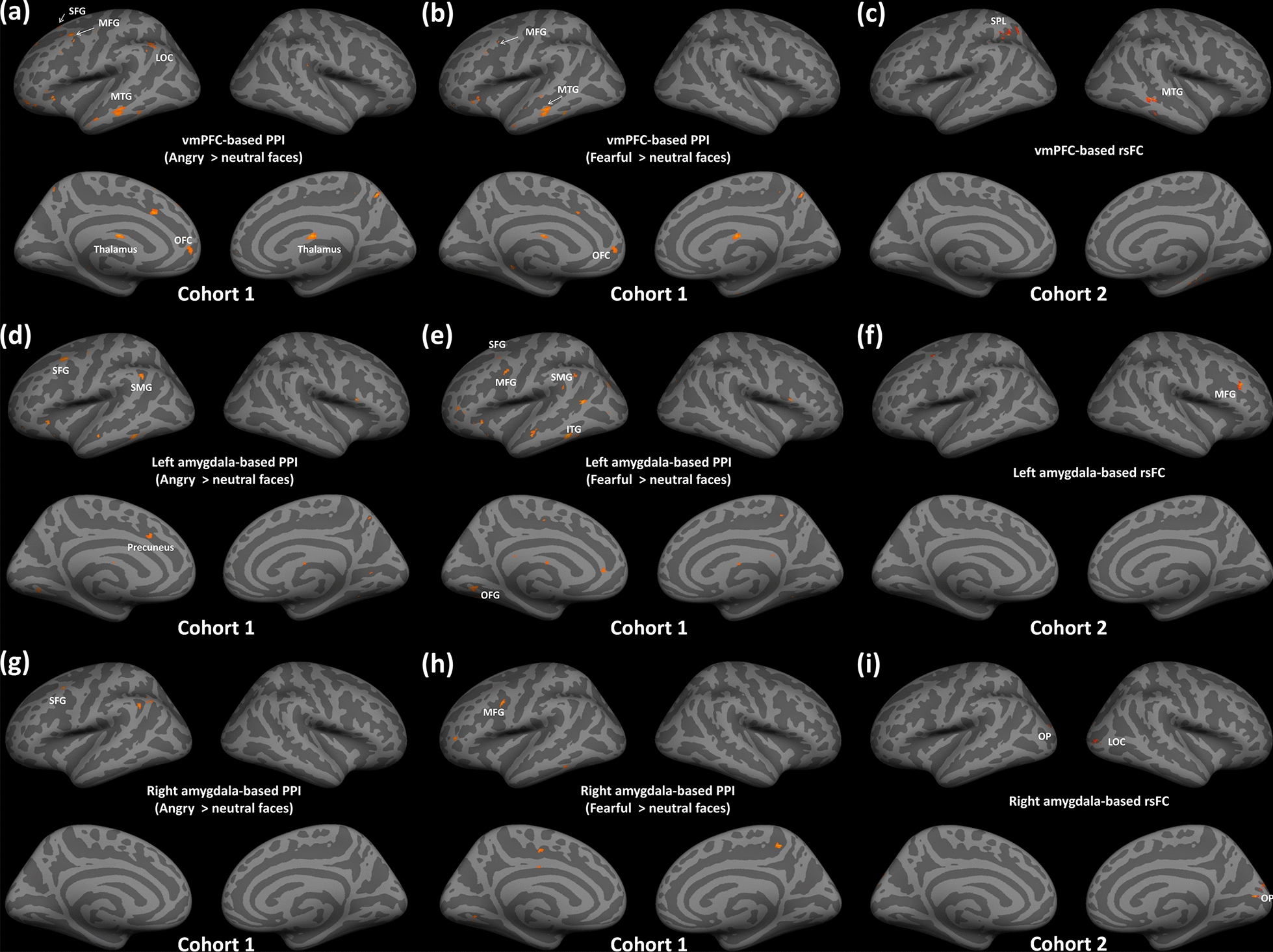
Results on the associations between task-based PPI and resting-state functional connectivity and GPT. Task-based PPI analyses indicated that compared to the group with low scores in GPT, the high group exhibited weaker functional connectivity when processing angry faces and fearful faces for vmPFC (**a**–**b**), left amygdala (**d**–**e**), and right amygdala (**g**–**h**) seeds in Cohort 1. Here, high (e.g., > median GPT score) and low (e.g., < median GPT score) group were defined based on the median score of GPT. Seed-based resting-state functional connectivity analyses showed positive correlation of GPT with vmPFC-based (**c**), positive correlation of GPT with left-amygdala-based (**f**), and negative correlation of GPT with right-amygdala-based functional connectivity (**i**) in Cohort 2

Given the importance of vmPFC on emotion regulation and generation, we selected the right vmPFC as the seed of interest for further task-based and resting-state functional connectivity analyses based on task-related findings. First, we focused on the task-based functional connectivity analysis with the widely used PPI approach in Cohort 1. The PPI analysis on the angry faces condition (e.g., Angry > Neutral faces) further revealed that compared to the low group, the high group exhibited weaker functional connectivity of the vmPFC seed with several brain regions, including the left SFG, bilateral MFG, left MTG, left OFC, left LOC, bilateral thalamus, and FP (see details in Table 3; Fig. 3a). For the fearful faces condition (e.g., Fearful > Neutral faces), the high group likewise indicated weaker functional connectivity of vmPFC with similar brain regions, including the left SFG, bilateral MFG, left OFC, and left MTG (see details in Table 3; Fig. 3b).

**Table 3:** Brain regions of task-based and resting-state FCs in vmPFC and GPT

Measure	Brian region	Cluster size (voxels)	MNI Coordinates			Z
			*x*	*y*	*z*	
vmPFC-based PPI High < Low group on the contrast of A > N (Cohort 1)	L Superior Frontal Gyrus	128	− 18	28	62	4.27
	L Middle Frontal Gyrus	71	− 36	8	64	4.19
	R Middle Frontal Gyrus	66	28	10	32	3.99
	L Middle Temporal Gyrus	55	− 66	− 22	− 16	3.64
	L Orbitofrontal Cortex	51	− 30	34	− 2	4.18
	L Lateral Occipital Cortex	47	− 52	− 60	40	3.68
	R Thalamus	45	12	− 8	18	4.03
	L Thalamus	43	− 8	− 8	14	4.11
	L Frontal Pole	37	− 36	46	− 6	3.71
vmPFC-based PPI High < Low group on the contrast of F > N (Cohort 1)	L Superior Frontal Gyrus	80	− 18	28	62	4.07
	R Middle Frontal Gyrus	75	28	16	30	4.20
	L Orbitofrontal Cortex	49	− 30	34	− 2	3.93
	L Middle Frontal Gyrus	35	− 44	16	36	3.79
	L Middle Temporal Gyrus	34	− 62	− 24	− 14	3.79
vmPFC-based rsFC Positive correlation (Cohort 2)	L Angular	84	− 40	− 54	52	3.61
	R Middle Temporal Gyrus	34	58	− 36	− 6	3.54
	L Superior Parietal Lobule	28	− 26	− 50	44	3.43
	R Parahippocampus	28	32	− 30	− 18	4.27
	L Middle Frontal Gyrus	25	− 32	− 2	62	3.30

Task-based PPI analyses revealed weaker functional connectivity of vmPFC-seed with several brain regions on the contrast of angry > neutral faces and fearful > neutral faces in high group compared to group with low scores of GPT in Cohort 1. Resting-state functional connectivity analyses showed positive correlations between GPT and vmPFC-seed FC with several regions in Cohort 2

*L* Left, *R* Right, *F* Fearful face, *A* Angry face, *N* Neutral face, *vmPFC* ventromedial prefrontal cortex

In Cohort 2, the rs-FC analysis further showed that GPT was significantly and positively correlated with the FC of the vmPFC seed with prefrontal-parietal cortex and ventral visual pathway, including the left angular, right MTG, left SPL, right parahippocampus, and left MFG (Table 3; Fig. 3c). We did not observe any negative association between the vmPFC-related FC and GPT.

### Bilateral amygdala-related task-based and resting-state FC and GPT

Although amygdala reactivity to negative emotional faces was not associated with GPT, we hypothesized that amygdala-related functional connectivity might be critical to understanding the underlying mechanisms of GPT. First, we defined the bilateral amygdala as the seed regions based on the contrast of fearful + angry faces > shapes in Cohort 1. Second, we conducted task-based PPI in Cohort 1 and resting-state functional connectivity analyses in Cohort 2. In Cohort 1, task-based PPI analyses on the angry face condition (e.g., Angry > Neutral faces) revealed that compared to the low group, the high GPT group exhibited weaker left amygdala functional connectivity, especially with the left MFG, left SFG, left precuneus, and left SMG (see details in Table 4; Fig. 3d). On the fearful faces condition (e.g., Fearful > Neutral faces), we likewise observed similar patterns, manifesting weaker left amygdala functional connectivity with the left FP, left SFG, left MFG, left SMG, left OFG, left Lingual, and left ITG on the high group (see details in Table 4; Fig. 3e).

**Table 4:** Brain regions of task-based and resting-state FCs in amygdala and GPT

Condition	Brain region	Cluster size (voxels)	MNI Coordinates			Z
			*x*	*y*	*z*	
lAmygdala-based PPI High < Low group on the contrast of A > N (Cohort 1)	L Middle Frontal Gyrus	93	− 38	8	62	4.10
	L Superior Frontal Gyrus	63	− 8	26	66	4.16
	L Precuneus	39	− 14	− 60	50	4.08
	L Supramarginal Gyrus	31	− 50	− 48	32	4.54
lAmygdala-based PPI High < Low group on the contrast of F > N (Cohort 1)	L Frontal Pole	53	− 42	46	6	3.52
	L Superior Frontal Gyrus	44	− 14	26	64	4.26
	L Middle Frontal Gyrus	43	− 38	8	62	3.96
	L Supramarginal Gyrus	39	− 50	− 48	32	4.01
	L Occipital Fusiform Gyrus	32	− 32	− 66	− 10	3.6
	L Lingual	31	− 14	− 70	− 8	3.63
	L Interior Temporal Gyrus	30	− 58	− 46	− 18	3.94
lAmygdala-based rsFC Negative correlation (Cohort 2)	R Middle Frontal Gyrus	57	50	28	28	4.03
	L Cerebellum	46	− 10	− 84	− 30	4.19
	R Cerebellum	31	8	− 86	− 30	3.78
rAmygdala-based PPI High < Low group on the contrast of A > N (Cohort 1)	L Superior Frontal Gyrus	44	− 12	18	68	3.88
	L Middle Frontal Gyrus	27	− 38	10	60	3.87
rAmygdala-based PPI High < Low group on the contrast of F > N (Cohort 1)	L Superior Frontal Gyrus	28	− 12	26	64	3.93
	L Supramarginal Gyrus	24	− 46	− 48	34	3.84
rAmygdala-based rsFC Positive correlation (Cohort 2)	L Lateral Occipital Cortex	40	− 26	− 88	24	3.52
	L Occipital Pole	36	− 10	− 90	24	3.63
	R Lateral Occipital Cortex	31	32	− 84	0	3.55
	R Occipital Pole	22	14	− 94	24	3.57

Task-based PPI analyses revealed weaker functional connectivity of left/right-amygdala-seed with several brain regions on the contrast of angry > neutral faces and fearful > neutral faces in high group compared to group with low scores of GPT in Cohort 1. Resting-state functional connectivity analyses showed positive correlation between GPT and right-amygdala-seed FC with several regions but negative correlation between GPT and left-amygdala-seed FC with several brains in Cohort *2*

*L and l* Left, *R and r* Right, *F* Fearful face, *A* Angry face, *N* Neutral face

For the right amygdala seed, task-based PPI analyses on the angry face condition revealed weaker functional connectivity of this seed with the left SFG and MFG on the high GPT group in Cohort 1 (Table 4; Fig. 3g). For the fearful face condition, the high group likewise showed weaker functional connectivity with the left SFG and SMG in Cohort 1 (Table 4; Fig. 3h).

Beyond the task-relevant FC analyses, in Cohort 2, rs-FC analyses further found that GPT was positively correlated with the functional connectivity between the left amygdala seed and the right MFG, and bilateral cerebellum (Fig. 3f), but negatively correlated with the right amygdala-seed-based functional connectivity with ventral visual pathway, including bilateral LOC, and bilateral OP (see details in Table 4; Fig. 3i).

## Discussion

This study explored the association between brain reactivity to emotional faces—with emphasis on the amygdala—and individual variability in GPT using task-based and resting-state fMRI across two independent cohorts. In Cohort 1, task-based fMRI analysis revealed significant correlations between GPT and brain reactivity to negative emotion faces (e.g., fearful and angry faces > shapes) in the vmPFC, but not the amygdala. Further, PPI analyses indicated that the high GPT group exhibited weaker functional couplings of the vmPFC-seed with a top-down control network and visual pathway during the processing of fearful or angry faces, compared to the low GPT group. In Cohort 2, resting-state functional connectivity analyses also showed positive associations between GPT and resting-state functional connectivity (rs-FC) between the vmPFC-seed and the top-down control network/visual pathway. This pattern was mirrored in the amygdala-seed-based FC with a top-down control network in both task-based PPI and resting-state fMRI analyses for the left amygdala. Additionally, the right amygdala exhibited negative rs-FC with a visual pathway. Our work provides a foundational investigation into the brain reactivity of high GPT individuals to negative emotional faces in the vmPFC, underscoring the importance of this region and its functional coupling with the top-down control/visual network.

Our task-based fMRI analyses in Cohort 1 found that the vmPFC’s reactivity to negative emotion faces—but not the amygdala’s—was negatively correlated with GPT variability. This aligns with prior studies highlighting the vmPFC’s morphological characteristic as key in predicting GPT using multivariate pattern analysis (MVPA) [103]. Both the amygdala and vmPFC have been shown to play vital roles in emotional face processing through top-down control (i.e., vmPFC and dlPFC) on amygdala reactivity, leading to emotional face perception, physical arousal, cognitive processing, and subjective emotion experience [31, 59, 67, 78, 95].. The amygdala performs several functions during emotional face processing, including emotional significance processing [68], automatic negative evaluation for facial emotion [15], explicit emotion discrimination [24], threat stimuli recognition [2], and facial feature coding and recognition [12]. Conversely, the vmPFC is argued to regulate negative emotional responses by inhibiting the amygdala, as evidenced by animal models, brain lesions, and electrophysiological studies [30, 52, 55, 88]. It has also been suggested to generate negative emotions [30].

We propose that greedy individuals may maintain typical threat-related emotion processing supported by the amygdala but might show reduced vmPFC-related generation and regulation of negative emotions. This viewpoint is backed by numerous questionnaire studies reporting increased negative affect and psychopathology among greedy individuals [89, 92, 93, 106]. Task-based PPI analyses showed that when processing negative emotion faces, greedy individuals in Cohort 1 tended to exhibit weaker functional connectivity between the vmPFC and the top-down control network/visual pathway. The top-down control network includes the prefrontal cortex (i.e., dlPFC), whereas the visual pathway comprises the MTG and LOC. Previous research has highlighted the role of the prefrontal cortex (e.g., dlPFC, MFG, SFG) in regulating negative emotions through attentional selection and reappraisal strategies [23, 49].

Given the vmPFC’s critical functions in generating and regulating negative emotions [31, 37, 65], its decreased functional coupling with the top-down control network could suggest two things. First, greedy individuals may exhibit muted fear-related responses due to dysregulation of the top-down control network, explaining their explicit demands and behavioral approach motivations to satisfy their desire for more. Second, they may have diminished emotion regulation ability due to weaker functional modulation from the prefrontal cortex. This, combined with the dissatisfaction inherent in greed, may explain why greedier individuals exhibit more negative emotion/affect and lower subjective happiness [106]. Furthermore, the visual network is crucial for processing socially and emotionally relevant visual stimuli like faces [1, 27, 28]. Decreased functional connectivity between the visual network and the vmPFC may explain altered emotional face processing and a disregard for others’ social and emotional information. Therefore, when encountering negative emotional stimuli, greedy individuals may not only show disrupted perceptual abilities for visual processing but also lack regulation from a top-down control network.

Resting-state functional connectivity analysis in Cohort 2 also found positive correlations between GPT and the rs-FC of the vmPFC-seed with the top-down control/visual pathway. The vmPFC is a key region for various functions, including autonomic and endocrine regulation [87, 97], fear conditioning and extinction [9], prospection [91], decision-making valuation [35, 100, 101, 104], and mentalization [74, 86]. Enhanced functional coupling of this region with a top-down control network could indicate increased cognitive modulation on vmPFC-related functions. This includes autonomic and endocrine control, negative emotion generation and regulation, and mentalization for others. Such modulation could help greedy individuals reduce maladaptive behaviors and negative emotional experiences.

The vmPFC is a hub region of the default mode network (DMN), which reflects a default mode of brain function when an individual is awake but not actively involved in attention-demanding or goal-directed tasks [5, 46]. DMN activity patterns may describe abstract features of ongoing mental content, integrated from across other cortical regions [79]. DMN is sensitive to individual differences in interpretation [111], including trait-level personality differences [43, 47] and perception of the world [6, 66]. Failure to suppress DMN nodes (e.g., vmPFC) can lead to negative internal thoughts among individuals with certain psychiatric disorders [75, 107]. Greedy individuals may require stronger functional interactions between the vmPFC and top-down control networks to reduce negative affect and potential mental disorders. Greedy individuals also exhibit specific visual processing supported by the visual pathway, with outputs sent to the vmPFC for high-level integration. This pattern suggests that greedy individuals may have altered their visual processing of negative emotional stimuli, leading to atypical emotional experiences and rumination. In conclusion, the visual-prefrontal circuit is crucial for understanding greed.

Although we found no significant correlation between amygdala reactivity to negative emotion faces and GPT, task-based PPI analysis showed weaker functional interactions between the amygdala and top-down control network in greedy individuals processing negative faces. Both brain regions are known to be involved in emotional perception and regulation, with the top-down control network inhibiting amygdala reactivity to negative stimuli [7, 39, 77]. Altered functional connectivity between the amygdala and top-down control network has been observed in emotion-related psychiatric disorders such as depression [13, 16, 36], anxiety [20, 39], and posttraumatic stress disorder [81, 82]. We speculate that the core neural mechanisms underlying the greed personality trait may involve atypical emotion regulation, despite normal emotional processing.

In Cohort 2, resting-state fMRI analysis revealed that GPT was positively and negatively correlated with left amygdala-prefrontal FC and right amygdala-visual pathway FC, respectively. This aligned with previous studies indicating lateralization in human facial emotion expression [57, 70]. Emotional processing is believed to generate stronger brain activity in the right hemisphere, regardless of modality [29, 44]. These asymmetric brain functions in the amygdala’s distinct hemispheres could reflect the complexity of dispositional personality formation and underscore the importance of the amygdala-visual-prefrontal circuit in shaping human personality.

## Limitations

Several considerations are noteworthy in this study. First, although parental education level was controlled for, the subjects’ educational attainment is a factor for future studies to consider as a potential correlate of dispositional personality. Second, our sample consisted of only university students, limiting the generalizability of our findings. Notably, we observed age-specific developmental patterns that warrant further exploration with a wider age range. Third, although the face-matching task is efficient for early studies to establish basic foundations in theory, it is limited in its representation of the complexity of real-world face recognition and emotional processing. Future research can utilize other tasks that elicit both domain-general and domain-specific negative emotions to broaden the scope of our findings. Fourth, our explanations related to amygdala reactivity and the top-down control network may only partially reflect human emotion processing given its simplistic conceptualization. Future studies could consider utilizing additional tasks that elicit emotions not limited to the amygdala and the top-down control network to provide a more comprehensive understanding of greed. Lastly, the correlational design of our study does not allow for inferring causality. Future research could employ experimental or quasi-experimental designs to derive stronger causal inferences and validate our findings.

## Conclusion

We found that vmPFC reactivity to negative faces and functional coupling with the top-down control/visual pathway are key to understanding the formation of this personality trait. Additionally, GPT may alter amygdala-related functional connectivity with these networks, showing distinct patterns with the vmPFC region. These insights significantly enhance our understanding of greed from an emotional processing perspective and underscore the specific roles of the vmPFC in this personality. The observed alterations in functional connectivity could potentially serve as neural markers for identifying highly greedy individuals, potentially opening new avenues for targeted interventions. Furthermore, the distinct patterns of amygdala-related functional connectivity among high GPT individuals may offer a unique window into the neural underpinnings of greed and its impact on emotional processing. This could pave the way for future research exploring the intricate interplay between personality traits, emotional processing, and brain function. In essence, our findings highlight the need for a more nuanced understanding of the complex interplay between personality, emotion, and brain function.

## Data Availability

The datasets used and/or analyzed during the current study are available from the corresponding author upon reasonable request.
